# Risk factors associated with the severity of obstructive sleep apnea syndrome among adults

**DOI:** 10.1038/s41598-020-70286-6

**Published:** 2020-08-11

**Authors:** Xiaobo Zhou, Qiao Lu, Shaoping Li, Zhongyin Pu, Fang Gao, Bo Zhou

**Affiliations:** Department of Psychosomatics, Sichuan Academy of Medical Sciences and Sichuan Provincial People’s Hospital, Chengdu, China

**Keywords:** Diseases, Medical research, Risk factors

## Abstract

Obstructive sleep apnea syndrome (OSAS) is a complete or partial airway obstruction that causes significant physiologic disturbance with various clinical impacts. The aim of the study was to determine the risk factors associated with the severity of OSAS. 303 patients with OSAS and 199 patients without OSAS enrolled in the sleep disorder center from 2017 to 2019 were included in the study. All patients completed physical examination, Epworth sleepiness scale, and polysomnography. By apnea–hypopnea index (AHI), patients were separated into four subsets: AHI < 5 as non-OSAS group, AHI ≧ 5 and < 15 as mild group, AHI ≧ 15 and < 30 as moderate group and AHI ≧ 30 as severe group. AVONA analyses, chi-square test, univariate and multivariate linear regression analyses were conducted to assess the correlation between specific factors and AHI. Our study demonstrated that patients with severe OSAS were associated with higher body mass index, higher systolic blood pressure awake in the morning, lengthened latent period of slow wave sleep, lower level of average oxygen saturation and minimum oxygen saturation (*P* < 0.05). Our findings provide evidence regarding several potentially useful factors for recognizing OSAS in adults, and physicians should promote the early recognition, diagnosis and intervention of OSAS.

## Introduction

Obstructive sleep apnea syndrome (OSAS) is one of the most common sleep-disorder breathing (SDB) conditions^1^. It is characterized by repeated episodes of upper airway obstruction resulting in cessation (apnea) or reduction (hypopnea) in airflow during sleep. It has been estimated that at least 4% adults in general population in China and even 19–40% middle-aged general population in the United States have some degree of OSAS^2,3^.

OSAS is associated with a number of adverse outcomes including hypertension, cardiovascular disease, stroke, diabetes, and motor vehicle accidents^4,5^. Research indicates that early recognition of OSAS will not only reduce morbidity but will also lead to a significant reduction in health care costs for other conditions^6^. To develop better strategies for the screening and treatment of OSAS, it is important to identify risk factors for OSAS.

Aging, male, obesity, body position, hypertension, abnormal Epworth sleepiness scale (ESS) scoring, lower oxygen saturation, and decreased slow wave sleep (SWS) were considered important factors of OSAS^7,8^. There were also studies indicating that OSAS was associated with several controversial risk factors. However, numerous other studies did not support these findings. Such discrepancies might be due to race and age differences among various study populations and varying definitions of OSAS. In addition, most of the study population had a relatively small sample size or narrow age ranges of childhood or adolescence, they failed to systematically assess a wide range of risk factors that were associated with OSAS.

The objective of our study was to identify potential risk factors of OSAS in adults in a clinical setting. Our study enrolled adults of a broad range of ages and included evaluation of potential risk factors of the severity of OSAS derived from previous published studies and from our own experience. We hypothesized that patients with severe OSAS were correlated with older age, male, obesity, supine position, hypertension, higher ESS scoring, lower oxygen saturation and decreased SWS.

## Results

The baseline characteristics were presented in Table 1. A total of 502 patients were enrolled in the study, including 303 patients with OSAS and 199 patients without OSAS. In the patients with OSAS, there were 69 (22.8%) females and 234 (77.2%) males. The age range was 19–83 years old and the average age was 44.6 years (SD = 12.4). The mean body mass index (BMI) was 25.7 ± 3.6 kg/m^2^. The mean ESS scoring was 8.6 ± 5.9, and the mean blood pressure was 123/74 mmHg before sleep and 123/76 mmHg awake in the morning. Mild OSAS group consisted of 90 patents, moderate OSAS group consisted of 103 patients and severe OSAS group consisted of 110 patients.

**Table 1:** Demographics and characteristics of all the participants by univariate analyses.

Variables	Mild OSAS (n = 90)	Moderate OSAS (n = 103)	Severe OSAS (n = 110)	Total/average	Non-OSAS (n = 199)	Statistics	*P* value
**Gender**							
Male	66 (73.3)	76 (73.8)	92 (83.6)	234 (77.2)	67 (33.7)	χ^2^ = 94.929	0.000**
Female	24 (26.7)	27 (26.2)	18 (16.4)	69 (22.8)	132 (66.3)		
**Age (years)**							
Mean (SD)	42.5 (13.0)	46.7 (12.1)	44.4 (11.9)	44.6 (12.4)	42.7 (13.5)	F = 2.715	0.100
Range	21–76	21–74	19–83	19–83	19–82		
**ESS scoring**							
Mean (SD)	7.3 (5.4)	7.8 (4.5)	10.3 (6.8)	8.6 (5.9)	6.3 (5.3)	F = 17.295	0.000**
**Height (cm)**							
Mean (SD)	166.2 (8.7)	166.7 (7.6)	168.3 (6.6)	167.1(7.6)	162.4 (7.9)	F = 44.156	0.000**
**Weight (kg)**							
Mean (SD)	66.0 (10.2)	71.3 (13.9)	77.1 (10.8)	71.8 (12.6)	57.4 (10.2)	F = 180.658	0.000**
**BMI**							
Mean (SD)	23.8 (3.1)	25.5 (3.9)	27.2 (3.2)	25.7 (3.6)	21.6 (2.8)	F = 166.804	0.000**
**Blood pressure (mmHg)**							
*Before sleep*							
SBP	120.0 (14.9)	120.8 (14.4)	129.5 (14.5)	123.7 (15.1)	114.6 (14.6)	F = 43.917	0.000**
DBP	72.4 (11.6)	72.2 (10.7)	79.3 (11.3)	74.8 (11.6)	68.4 (9.9)	F = 40.016	0.000**
*Awake in the morning*							
SBP	118.4 (14.6)	120.0 (13.9)	129.7 (15.1)	123.0 (15.4)	111.8 (15.1)	F = 60.926	0.000**
DBP	73.3 (10.7)	73.7 (10.5)	82.0 (11.5)	76.6 (11.6)	67.7 (10.3)	F = 71.150	0.000**

*SBP* systolic blood pressure, *DPB* diastolic blood pressure, *BMI* body mass index, *ESS* Epworth sleepiness scale.

**P value < 0.05.

Non-OSAS group consisted of 199 patients. The age range was 19–82 years old and the average age was 42.7 years (SD = 13.5). The mean BMI was 21.6 ± 2.8 kg/m^2^. The mean ESS scoring was 6.3 ± 5.3. The mean blood pressure was 114/68 mmHg before sleep and 111/67 mmHg awake in the morning.

Univariate and multivariate linear regression analyses were conducted to determine the risk factors associated with the severity of OSAS according to the apnea–hypopnea index (AHI) (Tables 2, 3).

**Table 2:** Factors associated with the severity of OSAS among all the participants by univariate linear regression analyses.

Variables	Standardized coefficients beta	Adjusted R square	Statistics value	*P*
Gender	0.345	0.117	8.206	0.000**
Age	0.001	− 0.002	0.018	0.986
ESS scoring	0.278	0.076	6.449	0.000**
Height	0.255	0.063	5.889	0.000**
Weight	0.523	0.272	13.693	0.000**
BMI	0.520	0.269	13.606	0.000**
SBP before sleep	0.324	0.103	7.628	0.000**
DBP before sleep	0.312	0.096	7.309	0.000**
SBP awake in the morning	0.394	0.153	9.360	0.000**
DBP awake in the morning	0.411	0.167	9.852	0.000**
**Latent period**				
N1	− 0.157	0.023	− 3.555	0.000**
N2	− 0.084	0.005	− 1.889	0.059
SWS	0.172	0.028	3.912	0.000**
REM	− 0.116	0.011	− 2.611	0.009**
**Duration**				
REM	0.005	− 0.002	0.115	0.908
NREM	0.035	− 0.001	0.788	0.431
SWS	− 0.157	0.023	− 3.555	0.000**
TST/TIB (%)	0.089	0.006	1.995	0.047**
**SpO**_**2**_** (%)**				
Average	− 0.435	0.187	− 10.791	0.000**
Minimum	− 0.739	0.545	− 24.529	0.000**
Supine duration	0.099	0.008	2.234	0.026**

*SBP* systolic blood pressure, *DPB* diastolic blood pressure, *BMI* body mass index, *ESS* Epworth sleepiness scale, *TST* total sleep time, *TIB* time in bed.

**P value < 0.05.

**Table 3:** Factors associated with the severity of OSAS among all the participants by multivariate linear regression analyses.

Variables	Beta	Statistics *t* value	*P*	95% CI
Gender	0.005	0.114	0.909	(− 3.904, 4.835)
ESS scoring	0.055	1.866	0.063	(− 0.012, 0.479)
Height	0.066	1.652	0.099	(− 0.037, 0.432)
Weight	− 0.101	− 0.328	0.743	(− 1.249, 0.891)
BMI	0.167	4.937	0.000**	(0.627, 1.456)
SBP before sleep	− 0.042	− 0.840	0.401	(− 0.220, 0.088)
DBP before sleep	0.041	0.876	0.381	(− 0.109, 0.285)
SBP awake in the morning	0.135	2.622	0.009**	(0.052, 0.360)
DBP awake in the morning	0.006	0.119	0.905	(− 0.193, 0.217)
N1 latent period	− 0.101	− 0.328	0.743	(− 1.249, 0.891)
SWS latent period	0.074	2.491	0.013**	(0.005, 0.040)
SWS duration	− 0.018	− 0.624	0.533	(− 0.060, 0.031)
TST/TIB (%)	− 0.017	− 0.550	0.583	(− 0.155, 0.087)
**SpO**_**2**_** (%)**				
Average	− 0.065	− 2.011	0.045**	(− 1.829, − 0.021)
Minimum	− 0.539	− 14.665	0.000**	(− 1.228, − 0.938)

*SBP* systolic blood pressure, *DPB* diastolic blood pressure, *BMI* body mass index, *ESS* Epworth sleepiness scale.

**P value < 0.05.

According to statistics, patients with severe OSAS were associated with higher BMI (*P* < 0.05, 95% CI 0.627–1.456), higher systolic blood pressure (SBP) awake in the morning (*P* < 0.05, 95% CI 0.052–0.360), lengthened latent period of SWS (*P* < 0.05, 95% CI 0.005–0.040), lower level of average oxygen saturation (*P* < 0.05, 95% CI − 1.829 to − 0.021) and minimum oxygen saturation (*P* < 0.05, 95% CI − 1.228 to − 0.938).

## Discussion

Our study indicated that BMI was a clinical predictor of the AHI. The correlation between BMI and OSAS was complex. Most of the literature in the past demonstrated that an increase in BMI was related to an increase in AHI^9,10^, although several studies did not find a significant relationship between BMI and AHI, supporting that truncal obesity was a better factor than BMI as predictors for OSAS^10^. The World Health Organization (WHO) defines ‘obesity’ as BMI ≧ 30 and this cutoff point provides a benchmark for individual assessment^11^. Obesity predisposed to OSAS, and the prevalence of OSAS was increasing due to the ongoing epidemic of obesity^12^. In addition, the average BMI for patients with severe OSAS was 27.2 kg/m^2^ in our study, indicating that overweight, although not obese was still a risk factor for severe OSAS.

Our study found that SBP awake in the next morning was a predicator of severe OSAS. Previous studies reported the link between OSAS and hypertension, and postulated mechanisms connecting elevated blood pressure to OSAS, including increased negative intrathoracic pressure increasing left ventricular transmural pressure^13^, increased sympathetic nervous system activation^14^, and autonomic nervous system dysfunction^15^. It is noteworthy that in our study, higher SBP level awake in the next morning was associated with higher AHI whereas diastolic blood pressure (DBP) level had no such association. The mechanism was still unclear and warranted further studies.

Surprisingly, our study found that lengthened latent period of SWS was associate with higher AHI. Few studies in the past reported the relationship between the severity of OSAS and latent period during the sleep except one study reported lengthened latent period of the rapid eye movement (REM) sleep in children with OSAS^16^. Therefore, more studies were warranted to clarify the relationship between AHI and latent period of sleep.

Our study found that low level of average oxygen saturation and minimal oxygen saturation during the sleep was associated with the severity of OSAS. Pulse oximetry is a relatively simple, feasible, and inexpensive method that has been extensively used to routinely assess patients’ oxygen saturation during sleep. However, the sensitivity and specificity of the oxygen saturation to predict OSAS varied in different studies^17^.

We also found that the duration of SWS was not significantly correlated with the severity of OSAS, which was inconsistent with previous findings reporting that AHI was significantly decreased during SWS^18^. Severe OSAS decreased occurrence of SWS and continuous positive airway pressure (CPAP) treatment of OSAS caused SWS rebound^19^. The different sample size and sample population may be a possible reason for the controversial conclusions and further studies need to be conducted.

Our study did not find the relationship between body position and severity of OSAS. The findings in the past literature were mixed. Upper airway collapsibility was greater in supine position compared to lateral position. In supine position, the tongue base narrowed the upper airway by the effect of gravity. Therefore, respiratory events were seen less in side position^20^. Over 60% of the patients with OSAS were considered to have a positional obstructive sleep apnea (POSA), and the degree of severity of OSAS was thought to be mostly associated with the sleep time spent in the supine position^21^. However, in other studies, total sleep time in supine position was not considered as a crucial factor of total AHI^22^ and differences in sleep position did not play a major role in night-to-night variability of AHI^23^. Therefore, it is still unclear how much variability in time spent supine contributes to total AHI and more research is warranted to clarity the issue.

Moreover, our study found that there was no significant correlation between ESS scoring and AHI, which was inconsistent with previous studies^24^. ESS was proven to be a reliable self-assessment tool, which could safely predict the presence of OSAS. It might be explained by the fact that although ESS has advantages of being brief and simple to carry out, it has also weaknesses related to subjective scoring.

The limitations of this research are pointed out. Firstly, this is a cross sectional study, therefore, we cannot make any certain conclusion about the correlation between these factors and AHI. In addition, the patients studied were from only one sleep disorder center, which could have resulted in sampling bias. Larger, multicenter studies are required to further investigate the risk factors for OSAS.

Our study demonstrated that the severity of OSAS was correlated with higher BMI, higher SBP awake in the morning, lengthened SWS latent period, lower level of average oxygen saturation and minimum oxygen saturation. Our findings provide evidence regarding several potentially useful factors for recognizing OSAS in adults, and physicians should promote the early recognition, diagnosis and intervention of OSAS.

## Methods

### Participants

A total of 303 adult patients with OSAS and 199 adult patients without OSAS were referred to sleep disorder center of department of psychosomatics, Sichuan Academy of Medical Sciences & Sichuan Provincial People’s Hospital from 2017 to 2019. All patients completed physical examination, ESS, and polysomnography (PSG).

The study was reviewed and approved by the Ethics Committee of Sichuan Academy of Medical Sciences and Sichuan Provincial People's Hospital, and conducted in accordance with the Declaration of Helsinki.

### Questionnaire

The most important scale for the assessment of daytime sleepiness is ESS published in 1991 by Murray Johns^25^. It consists of a self-administered questionnaire that investigates the extent of daytime sleepiness specifically and in a very simple manner. There are 8 items in ESS and subjects are asked to rate on a 4-point scale (0–3) his/her chances of dozing in each of 8 different situations that are often encountered in daily life. Arbitrarily, a score of ≧ 12 has been suggested as being abnormal and an indicator of excessive daytime sleepiness^25^.

### PSG data collection

The gold standard diagnostic method for OSAS is a full-night PSG^26^. The frequency of episodes of apnea and/or hypopnea per hour of sleep, also known as apnea–hypopnea index (AHI), as well as the lowest observed oxyhemoglobin saturation (O_2_SAT) during sleep is used as the main criteria for severity assessment. It is defined as non-OSAS for AHI < 5, mild for AHI ≧ 5 and < 15, moderate for AHI ≧ 15 and < 30, and severe for AHI ≧ 30^27^.

All patients had to have a minimum of 8 hours of monitored sleep in the sleep disorder center. They underwent nocturnal PSG monitoring (Philips Alice Version 6, Netherland) using 6 scalp electrodes (C3, C4, F3, F4, O1 and O2 locations), 2 reference electrodes behind the ears (left [A1] and right [A2] mastoid areas), 3 electromyographic electrodes over the submental muscles, 4 electromyographic electrodes over the leg muscles, 2 electro-oculographic electrodes, one ground electrode and nasal flow detector. No ingest alcohol or caffeine, nap, protracted or tedious exercises were informed during the study. Pulse oximeter was used to obtain nocturnal oximetry recordings. Electrocardiogram was used to obtain rhythm of the heart. The AHI documented the number of apnea-plus-hypopnea incidents every hour during sleep. Apnea could be viewed as being without respiration for more than 10 s. Hypopnea could be viewed as the deduction of at least 50% ventilation causing a drop in arterial saturation of at least 3%. OSAS could be viewed as apnea or hypopnea happening at least five times per hour, persisting for more than 10 s.

### Statistics

Statistical analyses were performed with SPSS 26.0 for mac. The summary of descriptive statistics was presented as mean (with SD) for continuous variables and as frequencies (with percentages) for categorical variables. The Pearson chi-square test was used for comparison of qualitative data. Univariate linear regression analyses were used to identify probable factors of severe OSAS. Statistically significant parameters in univariate linear regression analyses were further examined in a multivariate linear regression model. Statistically significant difference was considered if *P* value < 0.05.

### Ethics approval and consent to participate

The research was approved by the Ethics Committee of Sichuan Academy of Medical Sciences and Sichuan Provincial People's Hospital. Written informed consent was obtained from all participants.

### Informed consent

Written informed consent was obtained from all participants for the publication of any potentially identifiable images or data included in this article.

## Data Availability

The data used and analyzed during the current study are available from the corresponding author on reasonable request.
